# Climate change knowledge and associated factors among undergraduate sport sciences students: a single-center cross-sectional study at a Turkish university

**DOI:** 10.3389/fpubh.2026.1964700

**Published:** 2026-10-16

**Authors:** Cemile Nihal Yurtseven, Ali Kaya, Selcuk Bora Cavusoglu, Özge Ercan, Ahmet Uslu, Ayse Nilay Kantar

**Affiliations:** 1 Department of Sports Management, Faculty of Sports Sciences, Istanbul University-Cerrahpaşa, Istanbul, Türkiye; 2 Department of Recreation, Faculty of Sports Sciences, Istanbul University-Cerrahpaşa, Istanbul, Türkiye; 3 Istanbul University, Quality Coordination Office, Istanbul, Türkiye

**Keywords:** climate change literacy, environmental education, higher education, sport sciences, sustainability

## Abstract

Climate change is a major environmental and public health threat with consequences for human health, physical activity, and sport participation. Evidence concerning climate literacy among undergraduate sport sciences students remains limited. This study aimed to assess climate change knowledge and examine sociodemographic, academic, and information-source factors associated with knowledge levels among undergraduate sport sciences students. A cross-sectional online survey was conducted among sport sciences undergraduates. Based on a sample-size calculation for estimation of a population proportion, a minimum of 234 participants was required. A recruitment target of 300 accounted for potential non-response. All eligible students in the faculty (N ≈ 600) were invited, and 320 students comprised the final analytic sample. The questionnaire included sociodemographic, academic, and information-source variables together with a 20-item climate change knowledge test. Internal consistency was assessed using the Kuder–Richardson Formula 20 coefficient. Participant characteristics and knowledge scores were summarized descriptively. Associations between categorical variables and knowledge level were examined using Pearson’s chi-square tests. Multivariable multinomial logistic regression was used to identify factors independently associated with knowledge level, with the low-knowledge group as the reference category. Overall model significance and explanatory performance were assessed using the likelihood-ratio chi-square test and Nagelkerke’s pseudo-R^2^. The mean climate change knowledge score was 12.6 ± 3.4. Overall, 38.4% of participants were classified as having low knowledge, 41.6% as moderate knowledge, and 20.0% as high knowledge. Prior environmental education was associated with higher odds of high versus low knowledge (AOR = 2.34, 95% CI [1.28, 4.26], *p* = 0.006). Use of scientific or academic information sources (AOR = 1.89, 95% CI [1.07, 3.32], *p* = 0.029) and senior-year status (AOR = 1.76, 95% CI [1.01, 3.04], *p* = 0.045) were also associated with higher odds of high versus low knowledge. Climate change knowledge was predominantly moderate among participating students. The observed associations support further investigation of structured climate change education within sport sciences curricula. Because of the cross-sectional design, causal and temporal relationships cannot be established. Multicentre longitudinal studies and educational intervention trials are needed to confirm these associations and evaluate whether curriculum-based interventions improve climate literacy among sport sciences students.

## 1 Introduction

Climate change represents a major environmental and public health challenge with direct implications for sport and physical activity. Rising temperatures, air pollution, and extreme weather events may affect exercise safety, athlete health and performance, training continuity, outdoor recreation, and participation in sport. These climate-related exposures are particularly relevant to sport sciences because professionals in coaching, physical education, sport management, and recreation may increasingly be required to recognize environmental hazards and make informed decisions concerning athlete safety, physical activity, and climate-responsive sport practices. Therefore, adequate climate change knowledge represents an increasingly relevant competency for future sport professionals (1–3, 25–33).

University students constitute an important population in the societal response to climate change because their future professional and civic roles may influence mitigation, adaptation, and environmental sustainability. Previous studies indicate that university students generally recognize climate change as an important environmental problem; however, gaps and misconceptions may persist regarding its causes, consequences, and mitigation strategies (4, 5). Climate-related knowledge among university students may also vary according to educational experience, academic discipline, socioeconomic characteristics, and access to scientific and environmental information sources (6, 7). Higher education institutions therefore have an important role in developing climate-related knowledge and students’ capacity to make informed environmental decisions (8–10).

Despite increasing research on climate literacy among university students, evidence specifically concerning undergraduate sport sciences students remains limited. This represents an important population-specific gap because sport sciences graduates may work as coaches, physical education teachers, sport managers, and recreation professionals whose professional decisions can be affected by heat exposure, air quality, extreme weather, and other climate-related hazards (8–10). Evidence addressing climate change knowledge and its associated factors among sport sciences students in Türkiye also appears to be limited. Consequently, it is important to determine not only the level of climate change knowledge in this population but also the academic, educational, and information-related characteristics associated with differences in knowledge (11, 12).

The present study was informed by the climate literacy framework, which conceptualizes climate literacy as encompassing knowledge of the climate system, the ability to evaluate scientific information, and the capacity to make informed personal and professional decisions concerning climate change (13). Because the instrument used in this study primarily measures factual knowledge, the present analysis focuses specifically on the knowledge component of climate literacy rather than the full multidimensional construct. The explanatory variables were selected to represent educational, academic, informational, and demographic factors potentially related to climate knowledge. Academic program reflects differences in disciplinary exposure, year of study represents cumulative university-level educational exposure, prior environmental education reflects formal learning opportunities, and primary information source represents differences in access to scientific climate information. Gender was included as a demographic characteristic examined in previous research on environmental knowledge and related environmental constructs.

Accordingly, this study aimed to assess climate change knowledge among undergraduate sport sciences students and to examine sociodemographic, academic, educational, and information-related factors associated with different levels of climate change knowledge. The following hypotheses were tested: H1a, climate change knowledge differs across academic programs; H1b, senior-year students have higher climate change knowledge than students in lower years of study; H2, students who have previously received environmental education are more likely to have higher climate change knowledge than those without previous environmental education; and H3, students who primarily use scientific or academic information sources are more likely to have higher climate change knowledge than students relying on other information sources. Given the cross-sectional design, these hypotheses concern statistical associations and do not imply causal relationships.

## 2 Materials and methods

This cross-sectional study was conducted among undergraduate students enrolled in the Faculty of Sports Sciences at Istanbul University-Cerrahpaşa, Türkiye. Data collection was conducted from 30 April to 31 May 2026 following ethics committee approval. The study was reported in accordance with the STROBE statement for cross-sectional studies.

The study population comprised approximately 600 undergraduate students enrolled in three academic degree programs: Physical Education and Sports Teaching, Coaching Education, and Sports Management. The minimum sample size was calculated *a priori* for estimation of a population proportion using a 95% confidence level, an expected proportion of 0.50, and a margin of error of 0.05. After applying the finite population correction for an undergraduate population of approximately 600 students, the minimum required sample size was 234. An operational recruitment target of 300 participants was set prospectively to accommodate potential non-response. Because the study was designed primarily to estimate climate change knowledge and explore its associated factors, no separate *a priori* power calculation was performed specifically for the multinomial logistic regression. All eligible undergraduate students in the faculty (N ≈ 600) were invited to participate through institutional communication channels, and the final analytic sample comprised 320 students. Given that the high-knowledge category included 64 participants, estimates from the multinomial model, particularly for this outcome category, were interpreted cautiously.

We included undergraduate students aged 18 years or older from the Faculty of Sports Sciences who gave their informed consent to participate. Participation was entirely voluntary, and no financial or academic incentives were provided.

The climate change knowledge questionnaire was adapted from the instrument reported by Martha et al. (14) for use in the present population of undergraduate sport sciences students. The adapted questionnaire assessed factual knowledge concerning the causes of climate change and greenhouse gases, environmental and health effects, sport-related effects, mitigation, adaptation, and environmental policies, and was administered in Turkish. Internal consistency of the 20 dichotomously scored knowledge items was assessed in the present sample using the Kuder–Richardson Formula 20 (KR-20), yielding a coefficient of 0.78. This coefficient was interpreted solely as evidence of internal consistency and not as evidence of content, construct, or criterion validity. A comprehensive psychometric re-validation of the adapted instrument was not undertaken in the present study. Accordingly, the knowledge scores should be interpreted with consideration of this measurement limitation, and further research should evaluate the content validity, construct validity, and other measurement properties of the adapted instrument specifically among sport sciences students. The complete 20-item questionnaire used in the present study is provided as Supplementary material 1 to facilitate transparency and future validation.

The knowledge questionnaire concerned causes of climate change and greenhouse gases, environmental and health effects, sport-related effects, mitigation, adaptation, and environmental policies. Each item had three response alternatives: “True,” “False” and “I do not know.” Correct answers were scored 1, while wrong and “do not know” answers were scored 0 with the total score ranging from 0 to 20. The total knowledge score ranged from 0 to 20.

Consistent with the scoring approach reported by Martha et al. (14), total knowledge scores were categorized as low (0–10 correct answers), moderate (11–15 correct answers), and high (16–20 correct answers). The source study described the corresponding categories as poor, moderate, and good; in the present study, the terms low, moderate, and high were used for reporting clarity while retaining the original score thresholds. These cut-offs were adopted *a priori* from the source study and were not empirically derived from the distribution of scores in the present sample. They should therefore be interpreted as descriptive categories used to facilitate comparison and analysis rather than as clinically, diagnostically, or psychometrically validated thresholds (8, 14, 15).

The data were gathered through an anonymous online questionnaire that was disseminated via the institutional communication channels and student associations. Before accessing the questionnaire, an information page with details about the study purpose, the voluntary nature of participation, confidentiality, and the right to withdraw was presented to participants. Reminder messages were issued while the data were being collected.

The demographic characteristics and scores of climate change knowledge for the participants were presented in descriptive statistics. Descriptive statistics for continuous variables were expressed as mean and standard deviation or median and interquartile range, as applicable; and categorical variables were expressed as frequency and percentage. Associations between categorical participant characteristics and climate change knowledge level were examined using Pearson’s chi-square test. Degrees of freedom and *p*-values were reported for each analysis, and Cramér’s V was calculated as a measure of effect size.

Because the knowledge outcome comprised three ordered categories (low, moderate, and high), ordinal logistic regression was initially evaluated. The proportional-odds assumption was assessed using the test of parallel lines and was found to be violated (*p* < 0.05). Therefore, multinomial logistic regression was used because it does not require the effects of the explanatory variables to be constant across cumulative outcome thresholds. The low-knowledge group was used as the reference category. Adjusted odds ratios (AORs) and 95% confidence intervals (CIs) were estimated for moderate versus low knowledge and high versus low knowledge.

Explanatory variables were selected *a priori* based on the conceptual framework and previous literature rather than on statistical significance in univariable analyses. Gender, academic program, year of study, prior environmental education, and main source of information were included as explanatory variables. Consistent with the study hypothesis concerning senior-year status, year of study was dichotomized for the multivariable analysis as senior-year students versus students in all other years. All explanatory variables were entered simultaneously into the model.

Multicollinearity was assessed using variance inflation factors (VIFs), with values below 5 considered to indicate the absence of problematic multicollinearity. The distribution of observations across outcome and predictor categories was examined for sparse or empty cells. Overall model significance was assessed using the likelihood-ratio χ^2^ test, and explanatory performance was summarized using Nagelkerke’s pseudo-R^2^. Incomplete data or duplicate questionnaires were discarded prior to analysis. Analyses were conducted in IBM SPSS Statistics for Windows version 27.0 and a two-sided *p*-value less than 0.05 was considered significant. Ethical approval was obtained from the Istanbul University-Cerrahpasa Social and Human Sciences Research Ethics Committee (Approval No. 2026/288; 29 April 2026). The study was conducted in accordance with the Declaration of Helsinki. Electronic informed consent was obtained from all participants before participation.

## 3 Results

Our final analytic sample comprised 320 undergraduate students (meanage = 21.3  ± 2.1, range  = 18–24 years). Among the participants, 170(53.1%) were male, and 150(46.9%) were female. There were 128 (40.0%) students in Coaching Education, 106(33.1%) in Sports Management and 86 (26.9%) in Physical Education and Sport Teaching. A total of 132 students (41.2%) stated they had received prior climate- or environment-related education (Table 1).

**Table 1:** Demographic characteristics of participants (*n* = 320).

Variable	Category	n	%
Gender	Male	170	53.1
	Female	150	46.9
Program	Coaching Education	128	40.0
	Sports Management	106	33.1
	PE Teaching	86	26.9
Environmental education	Yes	132	41.2
	No	188	58.8

Climate change knowledge scores ranged from 4 to 19 (possible range: 0–20), with a mean score of 12.6 ± 3.4, corresponding to a moderate overall level of climate change knowledge. Table 2 presents the distribution of climate change knowledge scores according to gender.

**Table 2:** Descriptive statistics of climate knowledge scores by gender.

Gender	Mean± SD	Median	Min-Max
Male	12.1 ± 3.5	12	4–19
Female	13.2 ± 3.1	13	5–19

The distribution of knowledge levels in relation to their score ranges are 123 students (38.4%) low, 133 students (41.6%) medium, and 64 students (20.0%) high level of knowledge. Table 3 shows the distribution of climate change knowledge levels.

**Table 3:** Distribution of climate change knowledge levels.

Knowledge level	n	%
Low	123	38.4
Moderate	133	41.6
High	64	20.0

Climate change knowledge level differed according to prior environmental education (Table 4). Among students who had received prior environmental education, 24.2% were classified as having low knowledge, 46.2% as having moderate knowledge, and 29.6% as having high knowledge. Among students without prior environmental education, the corresponding proportions were 48.4, 38.3, and 13.3%, respectively.

**Table 4:** Knowledge levels according to environmental education.

Knowledge level	Environmental education yes	Environmental education no
Low	32 (24.2%)	91 (48.4%)
Moderate	61 (46.2%)	72 (38.3%)
High	39 (29.6%)	25 (13.3%)

Pearson’s chi-square tests were used to examine associations between climate change knowledge level and participant characteristics. Prior environmental education was significantly associated with climate change knowledge level, χ^2^(2) = 23.18, *p* < 0.001, Cramér’s V = 0.269. The complete results of the categorical analyses are presented in Table 5.

**Table 5:** Association between knowledge level and selected variables.

Variable	χ^2^	df	*p*	Cramér’s V
Gender	3.14	2	0.21	0.070
Program	4.82	4	0.306	0.087
Year of study	9.82	4	0.08	0.124
Prior environmental education	23.18	2	<0.001	0.269
Information source	8.94	2	0.03	0.118

In the multivariable multinomial logistic regression model, low knowledge was used as the reference outcome category (Table 6). Prior environmental education was associated with higher odds of both moderate knowledge (AOR = 1.62, 95% CI [1.04, 2.52], *p* = 0.031) and high knowledge (AOR = 2.34, 95% CI [1.28, 4.26], *p* = 0.006) relative to low knowledge. Use of scientific or academic information sources was associated with higher odds of high knowledge (AOR = 1.89, 95% CI [1.07, 3.32], *p* = 0.029), whereas the association for moderate versus low knowledge did not reach statistical significance (AOR = 1.45, 95% CI [0.98, 2.14], *p* = 0.063). Senior-year status was associated with higher odds of moderate knowledge (AOR = 1.41, 95% CI [1.02, 1.95], *p* = 0.038) and high knowledge (AOR = 1.76, 95% CI [1.01, 3.04], *p* = 0.045) relative to low knowledge. Gender was not independently associated with knowledge category after adjustment for the other variables included in the model. The overall model was statistically significant (likelihood-ratio χ^2^ = 34.12, *p* < 0.001), with a Nagelkerke pseudo-R^2^ of 0.18.

**Table 6:** Multivariable multinomial logistic regression analysis of factors associated with climate change knowledge levels (Reference category: Low Knowledge).

Predictor	Moderate vs. Low Knowledge AOR (95% CI)	*p*	High vs. Low Knowledge AOR (95% CI)	*p*
Previous environmental education (Yes vs. No)	1.62 (1.04–2.52)	0.031	2.34 (1.28–4.26)	0.006
Scientific or academic information source (Yes vs. No)	1.45 (0.98–2.14)	0.063	1.89 (1.07–3.32)	0.029
Senior-year student (Yes vs. Other)	1.41 (1.02–1.95)	0.038	1.76 (1.01–3.04)	0.045
Female gender (vs. Male)	1.05 (0.71–1.55)	0.804	1.12 (0.68–1.86)	0.640

AOR, Adjusted Odds Ratio; CI, Confidence Interval. Model statistics: likelihood-ratio χ^2^ = 34.12, *p* < 0.001; Nagelkerke pseudo-R^2^ = 0.18.

## 4 Discussion

Climate change knowledge among the participating undergraduate sport sciences students was predominantly moderate, while more than one-third were classified as having low knowledge. The findings provided differential support for the study hypotheses. No statistically significant association was observed between academic program and climate change knowledge level; thus, H1a was not supported. Senior-year status was associated with higher odds of both moderate and high knowledge relative to low knowledge, providing support for H1b, although the estimate for high versus low knowledge was imprecise and its confidence interval was close to the null value. Prior environmental education was associated with higher odds of both moderate and high knowledge, supporting H2. Use of scientific or academic information sources was associated with higher odds of high knowledge but not moderate knowledge relative to low knowledge; therefore, H3 was only partially supported. Gender was not independently associated with knowledge category after adjustment for the other variables included in the model. These findings should be interpreted as associations rather than causal effects because of the cross-sectional design. Overall, the findings are consistent with previous research indicating that university students may demonstrate general awareness of climate change while retaining important gaps in factual knowledge concerning its causes, consequences, and mitigation strategies (4, 5, 16).

Findings indicate that simply providing climate-related information may not be sufficient to cultivate the level of knowledge necessary for the development of professional decision-making for evidence in the sport context. One potential reason is that young people take in information about climate through news and social media and that might inform awareness more generally but not necessarily understanding from a scientific perspective. However, our study did not assess quantity/quality or credibility of the media exposure, and hence, this reading should be considered preliminary. Earlier findings suggest that multiple exposures to climate information do not necessarily lead to correct understandings of complicated processes like greenhouse gas emissions, radiative forcing, and climate feedbacks (17, 18). Prior environmental education was associated with higher odds of both moderate and high climate change knowledge relative to low knowledge. This finding is consistent with previous research reporting associations between formal environmental education and climate-related knowledge or literacy (1, 8). However, the cross-sectional design does not establish that environmental education caused the observed differences in knowledge. Students with greater pre-existing interest in environmental issues may have been more likely to seek environmental education, creating the possibility of self-selection. Residual confounding by other educational, socioeconomic, or attitudinal characteristics also cannot be excluded. Accordingly, the present finding should be interpreted as an association rather than evidence of the effectiveness of environmental education.

Formal environmental education may contribute to climate literacy by strengthening scientific understanding, critical evaluation of evidence, and knowledge of mitigation and adaptation strategies. Previous studies have associated sustainability-oriented education with greater environmental knowledge and more favorable environmental attitudes and behaviors (8, 9, 11, 12, 19). The association observed in the present study is consistent with this literature; however, the cross-sectional design does not establish that prior environmental education caused higher knowledge. Students with greater pre-existing interest in environmental issues may have been more likely to seek environmental education, and residual confounding cannot be excluded. Longitudinal and intervention studies are therefore required to determine whether structured climate education improves knowledge among sport sciences students.

Use of scientific or academic information sources was independently associated with higher odds of high, but not moderate, climate change knowledge relative to low knowledge. One possible explanation is that scientific and academic sources may provide more structured and evidence-based climate information than some informal sources. However, the direction of this association cannot be determined from the present cross-sectional data. Students with greater pre-existing climate knowledge may be more likely to seek scientific or academic sources, while exposure to such sources may also contribute to knowledge acquisition. Moreover, the study did not assess the frequency, quality, specific type, or credibility of information exposure. Therefore, the present findings should not be interpreted as evidence that non-academic information sources necessarily contain misinformation. Nevertheless, the observed association highlights the potential relevance of information literacy and critical appraisal of climate-related information within higher education (17, 18).

Gender showed no independent correlation with climate change knowledge. However, women’s higher environmental concern or pro-environmental attitude, as reported in a few prior investigations, should be understood as different concepts from factual climate knowledge (20). Hence, the lack of a significant correlation in this study should not be considered as an indication that men and women are equally strong in their environmental attitudes, concern, and behavior as these variables were not assessed.

Senior-year status was independently associated with higher odds of both moderate and high climate change knowledge relative to low knowledge. This association may reflect greater cumulative exposure to university-level education or broader academic experiences among students in later stages of their studies. However, the estimate for high versus low knowledge was relatively imprecise, with the lower bound of the 95% confidence interval close to the null value (AOR = 1.76, 95% CI [1.01, 3.04]). The finding should therefore be interpreted cautiously. Moreover, the cross-sectional design does not establish that progression through university education increased climate change knowledge, as unmeasured differences between senior-year and earlier-year students may have contributed to the observed association (10).

Climate change knowledge is increasingly relevant to sport and exercise practice because heat, air pollution, and extreme weather can affect exercise safety, athlete health and performance, training, outdoor recreation, and sport-event management. Rising temperatures may increase the risk of dehydration, heat-related illness, and impaired exercise performance, while exposure to air pollution may adversely affect respiratory and cardiovascular health during physical activity (21–23). Climate-related changes in natural environments may also affect outdoor recreation and sport tourism (24). Future sport professionals therefore require the knowledge and competencies needed to recognize environmental hazards, provide evidence-informed guidance, and contribute to climate-responsive sport practices.

The findings may have implications for curriculum development within sport sciences education, although their single-centre context should be considered. Climate-related content could be integrated into existing curricula through topics such as heat-illness prevention, air-quality hazards during physical activity, sustainable sport-event management, environmentally responsible facility operation, and critical evaluation of climate-related information. However, the present observational findings do not demonstrate that such curricular interventions would improve knowledge or professional competencies. Educational intervention studies are needed to evaluate whether integrating these topics into sport sciences curricula improves climate knowledge, professional decision-making, and climate-responsive competencies (11, 12).

This study has several limitations. First, participants were undergraduate sport sciences students recruited from a single university through an online voluntary-response survey. The sample should therefore not be considered representative of all sport sciences students in Türkiye, and the generalizability of the findings to other universities, regions, or educational contexts is limited. Voluntary participation may also have introduced selection bias if students with greater pre-existing interest in climate or environmental issues were more likely to participate. Second, the cross-sectional design precludes establishing temporal or causal relationships, and reverse causation remains possible for associations such as those involving prior environmental education and use of scientific or academic information sources. Third, the self-administered questionnaire may have been affected by response and recall biases. Fourth, categorizing the 0–20 knowledge score into low, moderate, and high groups resulted in loss of information, and the adopted cut-offs were derived from the source study rather than independently validated in the present population. Fifth, although the adapted knowledge questionnaire demonstrated acceptable internal consistency (KR-20 = 0.78), a comprehensive psychometric re-validation was not undertaken; therefore, further evidence regarding its content validity, construct validity, and other measurement properties is required. Sixth, the original sample-size calculation was based on estimation of a population proportion rather than an *a priori* power calculation specifically for multinomial logistic regression. Estimates involving the smaller high-knowledge group (*n* = 64) should therefore be interpreted cautiously. Finally, potentially relevant variables such as socioeconomic status, environmental attitudes, general academic performance, and the frequency and quality of previous exposure to climate-related information were not assessed and may have contributed to residual confounding.

## 5 Conclusion

Among undergraduate sport sciences students participating in this single-centre study, climate change knowledge was predominantly moderate, with a substantial proportion classified as having low knowledge. Prior environmental education and senior-year status were associated with higher odds of both moderate and high knowledge relative to low knowledge, while use of scientific or academic information sources was associated with higher odds of high knowledge. Gender was not independently associated with knowledge category. These findings represent associations and should not be interpreted as evidence of causal effects. Given the single-centre, cross-sectional design and the measurement limitations of the adapted questionnaire, the findings should be generalized cautiously. Multicentre longitudinal studies and educational intervention trials are needed to determine whether structured climate change education can improve climate knowledge and climate-responsive professional competencies among sport sciences students.

## Data Availability

The original contributions presented in the study are included in the article/Supplementary material, further inquiries can be directed to the corresponding author.
