# Supplementary material for: Climate change knowledge and associated factors among undergraduate sport sciences students: a single-center cross-sectional study at a Turkish university
Source: Front Public Health. 2026 Oct 16;14:1964700. doi: 10.3389/fpubh.2026.1964700 (PMC13720985; doi:10.3389/fpubh.2026.1964700)
Supplement: Supplementary file 1 [file Table_1.docx]

**Supplementary File S1. Questionnaire on Climate Change Knowledge Level Among Adolescents**

**Ek Dosya S1. Ergenlerde İklim Değişikliği Bilgi Düzeyi Anketi**

| **No** | **Soru (Question)** | **Seçenekler (Options)** |
| --- | --- | --- |
| **1** | Aşırı iklim olayları (sel, heyelan, orman yangınları ve sıcak dalgaları gibi) ölüme yol açabilir. | a. Evet  b. Hayır  c. Bilmiyorum |
| **2** | İklim değişikliği çocukların ve ergenlerin sağlık kalitesini etkiler. | a. Evet  b. Hayır  c. Bilmiyorum |
| **3** | İklim değişikliğinin birincil nedeni insan faaliyetleridir. | a. Evet  b. Hayır  c. Bilmiyorum |
| **4** | Deng (humması) gibi bulaşıcı hastalıkların görülme sıklığı iklim değişikliği nedeniyle artmaktadır. | a. Evet  b. Hayır  c. Bilmiyorum |
| **5** | İklim değişikliği, ishal gibi gıda ve su kaynaklı hastalıkların görülme sıklığını artırabilir. | a. Evet  b. Hayır  c. Bilmiyorum |
| **6** | İklim değişikliği bireyin ruh sağlığını etkiler (örneğin strese neden olur). | a. Evet  b. Hayır  c. Bilmiyorum |
| **7** | **Çevre yanlısı davranış (pro-environmental behavior) ne anlama gelir?** | a. Eylemlerimizin doğa üzerindeki olumsuz etkisini en aza indirmek için yapılan bilinçli davranış  b. Eylemlerimizin doğa üzerindeki olumsuz etkisini en aza indirmek için yapılan bilinçsiz davranış  c. Eylemlerimizin doğa üzerindeki olumlu etkisini en aza indirme davranışı  d. Eylemlerimizin doğa üzerindeki olumsuz etkisini en üst düzeye çıkarma davranışı  e. Bilmiyorum |
| **8** | **Sera etkisinin tanımı nedir?** | a. Atmosferde sıcak havayı Dünyanın dışında tutan gaz  b. Dünyanın koruyucu ozon tabakasının zarar görmesi  c. Asit yağmuruna neden olan kirlilik  d. Bitkilerin nasıl büyüdüğü ve geliştiği  e. Bilmiyorum |
| **9** | **Küresel ısınmaya en çok ne katkıda bulunur?** | a. Arabalar ve kamyonlar  b. Isınma ve elektrik için fosil yakıtların yakılması  c. Ozon tabakasındaki delikler  d. Ormansızlaşma (ağaçların kesilmesi)  e. Bilmiyorum |

**İklim krizini çözmek için en etkili önlemler nelerdir?**

| **Kategori** | **Önlem / Politika** | **Seçenekler** |
| --- | --- | --- |
| **1. Ekonomi Politikaları** | İklim krizini çözmede ekonomi odaklı yaklaşımlar | a. Kâr ve çevresel sürdürülebilirliği dengeleyen yeşil işletmelere daha fazla yatırım yapmak  b. Bir ürünün üretimi hakkında eksiksiz bilgi verilmesini zorunlu kılmak  c. Sanayi sektörünü genişletmek  d. Şirketlerin ürettikleri kirlilik için ödeme yapmasını sağlamak  e. Bilmiyorum |
| **2. Ulaşım Politikaları** | İklim krizini çözmede ulaşım odaklı yaklaşımlar | a. Daha fazla elektrikli araba, motosiklet ve otobüs kullanmak  b. Ulaşımda yenilenebilir enerji kullanmak  c. Kent ve kır tasarımını/planlamasını geliştirmek  d. Yayalar için yeterli alan sağlamak  e. Bilmiyorum |
| **3. Toplumun Korunması** | Afet ve toplum sağlığını koruma yaklaşımları | a. Afet kurumlarını koordine etmek  b. Afetler için daha fazla erken uyarı sistemi kurmak  c. İyi ve uygun fiyatlı sigorta imkanı sağlamak  d. Canlıları ve yaşam alanlarını korumak için altyapı ve doğa koruma çalışmaları yapmak  e. Bilmiyorum |
| **4. Enerji Politikaları** | Enerji üretimi ve kullanımı odaklı yaklaşımlar | **a. Güneş, rüzgar ve diğer yenilenebilir enerjileri kullanmak**  b. Evlerde ve binalarda enerji kullanımını tasarruflu hale getirmek  c. Kirliliğe yol açan yakıtların yakılmasını durdurmak  d. Kömürlü termik santraller kurmak  e. Bilmiyorum |
| **5. Yanlış İnanç Değerlendirmesi** | Fosil yakıtları yakmayı durdurursak, atmosferdeki karbondioksit seviyesi hemen düşecektir. | a. Evet  b. Hayır  c. Bilmiyorum |

**Aşağıdakilerden hangisi bir fosil yakıttır?**

| **Yakıt Türü** | **Seçenekler** |
| --- | --- |
| **1. Petrol** | a. Evet / b. Hayır / c. Bilmiyorum |
| **2. Kömür** | **a. Evet** / b. Hayır / c. Bilmiyorum |
| **3. Doğal gaz** | **a. Evet** / b. Hayır / c. Bilmiyorum |
| **4. Odun** | a. Evet / **b. Hayır** / c. Bilmiyorum |
| **5. Hidrojen** | a. Evet / **b. Hayır** / c. Bilmiyorum |
| **6. Güneş Enerjisi** | a. Evet / **b. Hayır** / c. Bilmiyorum |

**Puanlama Kriteri (Scoring Criteria):**

**Doğru Cevap:** 1 Puan

- **Yanlış Cevap / Bilmiyorum:** 0 Puan
